# The Need of Handwash Station Resources in Health Education Institutions to Promote Standardized WHO Handwashing Models

**DOI:** 10.1155/ipid/2423211

**Published:** 2026-07-30

**Authors:** Hamsu Kadriyan, Yasmin Sabrina Zulkifli, Eka Arie Yuliyani

**Affiliations:** ^1^ Department of Otolaryngology Head and Neck Surgery, Faculty of Medicine, University of Mataram, Mataram, Indonesia; ^2^ Department of Undergraduate, Faculty of Medicine, University of Mataram, Mataram, Indonesia

**Keywords:** compliance, hand hygiene, handwash stations, higher education, WHO standards

## Abstract

**Background:**

Hand hygiene is the fundamental basis for infection prevention and control, particularly in health education settings where students are trained as future healthcare professionals. Despite adequate knowledge, suboptimal handwash station infrastructure has a potency to hinder adherence to WHO hand hygiene standards.

**Methods:**

A cross‐sectional single center study was conducted among 273 preclinical medical students. Validated questionnaires assessed hand hygiene awareness, compliance, and handwash station usage, supported by structured facility observations. Spearman’s rank correlation test was applied for bivariate analysis.

**Results:**

Most respondents demonstrated high awareness of hand hygiene (96.7%) and high compliance (85.0%). No significant association was observed between awareness and handwash station usage (*p* = 0.410). However, handwash station usage was significantly associated with hand hygiene compliance (*p* < 0.001). Observations showed that the primary handwashing infrastructure is sufficient, but ancillary facilities remain suboptimal.

**Conclusion:**

High awareness alone is insufficient to ensure compliance with WHO‐standardized hand hygiene practices. Adequate, standardized, and accessible handwash station resources play a critical role in translating knowledge into consistent hygienic behavior.

## 1. Background

Hand hygiene is recognized as the most effective and cost‐efficient measure for preventing healthcare‐associated infections and interrupting the transmission of diseases worldwide [1, 2]. The World Health Organization (WHO) emphasizes correct handwashing techniques and appropriate facility design as essential components of infection prevention and control programs [1–3]. In health education institutions, hand hygiene competency is a fundamental professional requirement, as students’ habitual practices during training influence future clinical behavior [4, 5]. Therefore, the early exposure to hand hygiene protocols must be prioritized in health‐related higher education setting.

Despite extensive educational efforts, a persistent gap between hand hygiene knowledge and actual practice has been reported across healthcare and educational settings especially in low and middle‐income countries (LMICs) [4, 5]. This discrepancy is frequently shaped by a complex interaction of psychological, social, and environmental determinants [6–8]. The health belief model (HBM) hypothesizes that health actions are driven by an individual’s evaluation of disease threats and the efficacy of preventive measures, encompassing six dimensions: perceived susceptibility, severity, benefits, barriers, cues to action, and self‐efficacy [9]. Furthermore, the theory of planned behavior (TPB) offers a highly pertinent framework, suggesting that behavior is mediated by behavioral intentions, which are fundamentally influenced by attitudes, subjective norms, and perceived behavioral control [10]. Infrastructure plays a critical role among these barriers; insufficient availability of sinks, lack of water or soap, and the use of manually operated faucets lower compliance and increase the risk of environmental pathogen transmission [11, 12]. Importantly, poorly designed sinks may also become reservoirs for pathogenic microorganisms that paradoxically increase the infection risk [12].

In Indonesia and other LMICs, inadequate hand hygiene infrastructure remains a major challenge in both healthcare facilities and health training institutions [12–14]. Preliminary observations at a health education institution in Lombok, Indonesia, revealed a predominant utilization of manually operated handwash stations, which do not comply with WHO recommendations for touchless systems. While understanding how these infrastructural conditions influence behavior is critical for designing effective preventive strategies, literature concerning hand hygiene infrastructure analysis within LMIC health education settings remains scarce.

To address this literature gap, this study aimed to evaluate the relationship of handwash station resources in promoting awareness and compliance with WHO‐standardized handwashing practices among health education students.

## 2. Methods

### 2.1. Study Design and Setting

This study integrates the HBM and TPB to explore how handwash station resources influence awareness and compliance of the student with standardized WHO handwashing practices. A quantitative cross‐sectional single center study was conducted at the Faculty of Medicine, University of Mataram, Indonesia, between 2024 and 2025.

### 2.2. Participants and Sampling Strategy

The study population comprised all active preclinical medical students from the 2022–2024 cohorts totaling 857 students. Sample size was calculated with Slovin’s formula with a 5% margin of error, and the minimum required sample size was calculated to be 273 participants. The participant was obtained through purposive sampling to ensure that the sample specifically consisted of medical students who routinely utilized the faculty’s campus facilities and handwash stations during their active preclinical training blocks. This approach was chosen to exclude students who were away from campus due to academic leave or external assignments, ensuring that respondents had direct, daily interaction with the infrastructure being evaluated.

To be included in the study, participants had to meet the following inclusion criteria: (1) being an active preclinical medical student registered in the 2022, 2023, or 2024 cohorts; (2) regularly attending on‐campus academic activities during the study period; and (3) voluntarily consenting to participate. Students were excluded if they met one of the following criteria: (1) submitting incomplete questionnaires; (2) having a known allergy to the soap ingredients; or (3) presenting with dermatological conditions on the hands that prevented them from washing their hands.

### 2.3. Instruments

Data collection employed three questionnaires developed by the authors based on established behavioral frameworks. To ensure content validity, the initial items were reviewed and approved by two academic advisors to confirm that the questions accurately represented the constructs of the HBM and the TPB. Subsequently, a pilot study was conducted with a subset representing 25% of the total sample size to establish construct validity and internal consistency. Construct validity was evaluated at the item level using Pearson’s product‐moment correlation coefficient, where an item was considered valid if the two‐tailed significance value was less than 0.05 (*p* < 0.05). Reliability was assessed using Cronbach’s alpha (*α*).

1. Handwashing Awareness Questionnaire (7 items): all 7 items developed based on the HBM met the construct validity criteria (*p* < 0.05), demonstrating strong internal consistency (*α* = 0.808).
2. Handwashing Compliance Questionnaire (6 items, TPB‐based): all 6 items were statistically valid (*p* < 0.05) with an acceptable reliability (*α* = 0.717).
3. Handwash Station Usage Questionnaire (14 items): all 14 items assessing accessibility, usability, and perceived support for hand hygiene were statistically valid (*p* < 0.05) with high reliability (*α* = 0.812).

In addition, structured observational checklists were used to assess the physical condition, accessibility, and educational support of handwash stations across classrooms and laboratories, including restrooms. To maintain consistency and eliminate inter‐rater variability, all physical observations were conducted by a single, trained investigator who was blinded to the specific questionnaire data of the student cohorts. The full items for all three questionnaires and the detailed observation checklist used in this study are available as Supporting Information (available here).

### 2.4. Data Analysis

Descriptive statistics were used for univariate analysis. Normality testing using Kolmogorov–Smirnov showed nonnormal data distribution if *p* < 0.05. Spearman’s rank correlation test was therefore applied for bivariate analysis, with a significance level set at *p* < 0.05.

### 2.5. Ethical Considerations

Ethical approval was obtained from the University Ethics Committee with number of approval letter 169/UN18.F8/ETIK/2024. All participants provided informed consent prior to data collection.

## 3. Results

### 3.1. Participant Characteristics

During the study period, a total of 273 students participated; 69.2% were female, and 30.8% were male. Students from the 2024 cohort constituted the largest proportion (40.3%) (Table 1).

**TABLE 1:** Characteristics of respondents.

Characteristics		Number	Percentage
Gender	Male	84	30.8
	Female	189	69.2

Entrance (years)	2022	77	28.2
	2023	86	31.5
	2024	110	40.3

Handwashing awareness	High	264	96.7
	Moderate	9	3.3
	Low	0	0

Handwashing compliance	High	232	84.9
	Moderate	40	14.7
	Low	1	0.4

Handwash station usage	High	129	47.3
	Moderate	138	50.5
	Low	6	2.2

### 3.2. Handwashing Awareness and Compliance

According to Table 1, a high level of handwashing awareness was observed in 96.7% of respondents (264 of 273), while 85.0% demonstrated high compliance with WHO standardized hand hygiene practices. On the other hand, the low level of awareness and compliance were extremely low (0% and 0.4% consecutively).

### 3.3. Handwash Station Usage

Approximately half of the respondents reported moderate usage of handwash stations (138 of 273 or 50.5%), while 47.3% reported high usage. Data obtained from direct observation of handwash stations are presented in the following section.

### 3.4. Correlation Analysis

Based on correlation analysis, no significant association was identified between handwashing awareness and handwash station usage (*p* = 0.410). In contrast, a statistically significant association was found between handwash station usage and handwashing compliance (*p* < 0.001) (Table 2).

**TABLE 2:** Correlation between handwashing awareness and handwashing compliance with handwash station usage.

Variables	Handwash station usage	
	Coefficient correlation (Spearman’s rho)	Significance level (*p*)
Handwashing awareness	0.05	0.41
Handwashing compliance	0.312	< 0.001^∗^

^∗^Significance level at 0.05.

### 3.5. Direct Observation Regarding the Physical Condition, Accessibility, and Educational Support of Handwash Stations

**FIGURE 1:**
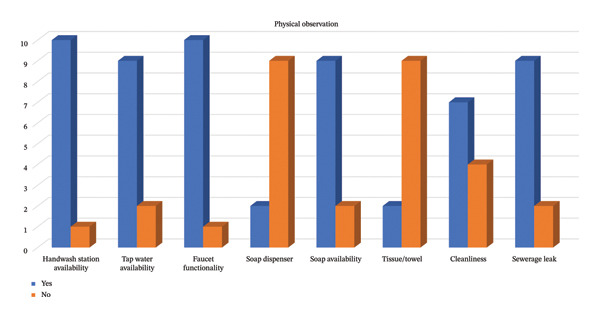
Physical observation of handwash stations.

Observation was done in 11 spots within the study site. The authors found that the physical condition of the handwash stations was adequate. Overall, these findings indicate that the physical infrastructure of the handwash stations is generally sufficient (65.9%). However, several ancillary facilities such as soap dispensers, hand dryers, and the sanitation of the surrounding area remain suboptimal (34.1%). The complete checklist evaluation result for all characteristics is seen in Figure 1.

**FIGURE 2:**
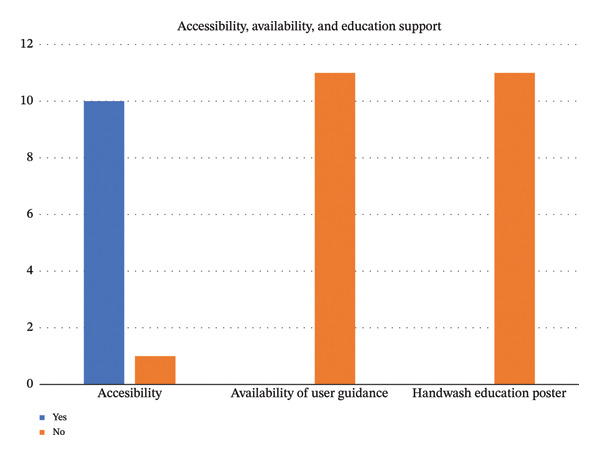
Handwash station accessibility, availability of handwashing guidance, and education support.

Regarding accessibility, the assessment recorded an excellent performance (90.9%). In contrast, the availability of hand hygiene guideline and educational support were absent. These findings suggest that while accessibility requirements are largely fulfilled, the guideline and educational support have not been implemented (Figure 2).

## 4. Discussion

This study underscores the critical urgency of evaluating not only the quantity and availability of handwashing unit but also their functionality, design, and accessibility within health education institutions. These factors significantly influence student perceptions regarding the benefits of and barriers to handwashing, in alignment with the HBM framework [9]. Furthermore, this study incorporates the TPB as a complementary behavioral approach, which asserts that students’ intentions to wash their hands are heavily influenced by their attitudes toward the behavior, subjective norms, and perceived behavioral control [10]. A positive attitude toward handwashing is fostered when students perceive facilities as clean, modern, and user‐friendly. Similarly, social norms within the academic environment are strengthened when infrastructure actively supports healthy behaviors. Furthermore, bolstering the perception of control through well‐placed, touchless facilities is likely to improve handwashing consistency [8, 12].

This study demonstrates that high awareness of hand hygiene among health‐related students (96.7%, *n* = 264) does not automatically translate into optimal compliance with WHO‐standardized handwashing practices. While a substantial proportion of students demonstrated high compliance (84.9%, *n* = 232), a critical segment exhibited only moderate to low compliance (14.7% moderate, *n* = 40; 0.4% low, *n* = 1). This stark disconnect highlights a profound intention–behavior gap. While cognitive awareness has been successfully established through health curricula and clean‐living campaigns, knowledge alone remains wholly insufficient to override environmental friction [15].

Our physical observation data directly substantiate this barrier, revealing critical institutional deficiencies that paralyze action. Out of the observed facilities, an overwhelming majority completely lacked essential consumables, with 9 out of 11 stations devoid of soap dispensers and 9 out of 11 stations entirely lacking tissues or paper towels. Furthermore, environmental and biological safety was severely compromised, as evidenced by 9 out of 11 stations presenting active sewerage leaks, alongside frequent deficits in baseline cleanliness (4 out of 11 units graded as unclean).

From the HBM perspective, poor facility maintenance (dirty sinks, empty soap, and leaks) serves as a critical “perceived barrier” that outweighs the known health benefits of handwashing, ultimately lowering compliance. Furthermore, a poorly maintained handwashing unit ceases to serve as a visual “cue to action.” Instead of prompting the habit, a neglected facility signals to the student that hand hygiene is not an institutional priority, thereby neutralizing their high academic awareness and halting the behavior before it begins. Consequently, interventions must pivot away from redundant educational campaigns and instead focus on structural engineering modifications and reliable supply chains that eliminate these immediate environmental barriers. Fudolig et al. found that the specific intervention should be done to specific profile of handwashing behavior [16].

The strong association between handwash station usage and compliance underscores the critical role of environmental support (*r*_*s*_ = 0.312; *p* < 0.001). According to the TPB framework, student’s perceived behavioral control is a key determinant of action, which is directly dictated by the accessibility, design, and hygiene of the available infrastructure. Interestingly, despite high general compliance figures, frequent handwash station usage was surprisingly low, with less than half of the cohort demonstrating high usage (47.3%, *n* = 129), while the majority fell into the moderate category (50.5%, *n* = 138). This suppressed utilization is heavily tied to the fact that while structural station availability (10 out of 11) and basic tap water supply (9 out of 11) were physically present, the structural quality was deeply flawed.

In this context, the predominance of manually operated station observed in this study is particularly concerning. Infrastructure that fails to meet WHO standards may not only limit compliance but also increase the risk of cross‐contamination at contact points [17]. Manually operated taps introduce a severe psychological risk: the certainty of recontaminating clean hands upon manually closing the valve. This structural flaw creates a negative feedback loop that decimates perceived behavioral control. Even when a student maintains a positive attitude and acknowledges strong subjective norms, the immediate friction of a manual contact point convinces them that executing standard hand hygiene is entirely outside of their convenient control [18]. The physical environment actively paralyzes psychosocial intent, shifting handwashing from an automated safety habit into an institutional chore.

These infrastructural limitations carry severe, long‐term implication within health educations setting, mirroring documented drivers of hand hygiene noncompliance among professional health workers [19, 20]. Students internalize habitual practices during training, which later shape professional behavior in clinical settings. Institutions that fail to provide standardized handwash station resources risk perpetuating suboptimal infection prevention practices among future healthcare cohorts [4, 5, 18].

However, these findings must be contextualized within the socioeconomic realities of higher education institutions in LMICs like ASEAN countries [21]. In these settings, institutional budgets frequently prioritize essential academic and clinical training equipment over advanced public health infrastructure. This manifests as a high prevalence of manually operated, basic wash stations rather than automated, touchless systems. Furthermore, systemic LMIC infrastructural vulnerabilities, as empirically verified by our observed water supply interruptions (2 out 11 stations lacked tap water) and severe supply chain delays for consumables, create an unreliable environment. Students are forced to constantly calculate whether a handwashing attempt is worth the logistical effort, ultimately choosing convenience over standard clinical protocol.

This study has several limitations, which should be taken into consideration when interpreting these finding. First, the cross‐sectional design limits the ability to infer causal relationships between handwash station resources, awareness, and compliance. Second, data on awareness and compliance were partly self‐reported, which may introduce social desirability bias despite the inclusion of observational components. Another limitation is that this study was conducted in a single institution, which may limit the generalizability of the findings to other health‐related higher education settings with different infrastructural or cultural contexts. Finally, microbiological assessment of handwash stations was not performed; therefore, conclusions regarding actual contamination risks were based on existing literature rather than direct measurement.

Further research should be done with longitudinal or interventional designs to evaluate cause‐and‐effect relationship between upgrading handwash station infrastructure and sustained hand hygiene compliance. Multicenter studies involving different universities and health training institutions are recommended to improve external validity. Additionally, future research should assess the effectiveness of integrating WHO’s multimodal hand hygiene strategy—combining infrastructure improvement, behavioral interventions, and continuous feedback—within health education curricula.

## 5. Conclusion

This study reveals that high cognitive awareness of hand hygiene guidelines does not guarantee the actual utilization of handwash station. Instead, behavioral compliance is heavily dependent on the physical engagement with these facilities. These findings demonstrate that institutional health programs require interventions that extend far beyond classroom‐based knowledge. While students frequently possess excellent theoretical awareness, their actual compliance is dictated by the availability, accessibility, and operational quality of handwashing infrastructure. Suboptimal, manually operated facilities act as structural barriers that hinder compliance and increase the risk of environmental cross‐contamination. Consequently, strategic institutional investments in automated, touchless, and well‐positioned handwash stations are essential to successfully transform student awareness into consistent hygienic habits, ultimately fostering a robust culture of infection prevention among future healthcare professionals.

## Author Contributions

Hamsu Kadriyan and Yasmin Sabrina Zulkifli designed the research; Yasmin Sabrina Zulkifli collected the data; and Hamsu Kadriyan, Yasmin Sabrina Zulkifli, and Eka Arie Yuliyani developed the manuscript.

## Funding

This research was supported by the University of Mataram (Grant No. 1392/UN18.L1/PP/2024).

## Disclosure

All authors agree with the final manuscript.

## Conflicts of Interest

The authors declare no conflicts of interest.

## Supporting Information

Additional supporting information can be found online in the Supporting Information section.

## Supporting information


**Supporting Information 1** Questionnaire and observation checklist.

**Supporting Information 2** STROBE statement—checklist of items that should be included in reports of cross‐sectional studies.

## Data Availability

Data are available on request from the authors.
