# Supplementary material for: The Need of Handwash Station Resources in Health Education Institutions to Promote Standardized WHO Handwashing Models
Source: Interdiscip Perspect Infect Dis. 2026 Jul 30;2026:2423211. doi: 10.1155/ipid/2423211 (PMC13421920; doi:10.1155/ipid/2423211)
Supplement: Supplementary file 1 — Supporting Information 1 Questionnaire and observation checklist. [file IPID-2026-2423211-s002.pdf]

Observation Checklist

Research Title:

THE CORRELATION BETWEEN HAND-WASH STATION USAGE, HANDWASHING AWARENESS, AND HANDWASHING COMPLIANCE AT THE FACULTY OF MEDICINE AND HEALTH SCIENCES, UNIVERSITY OF MATARAM

Observer Name : ____
Observation Date : ____
Observation Location : ____
Observation Time : ____

A. Physical Condition of the Hand-Wash Station

No.	Observation Items	Checklist	Notes
1	Hand-wash station is available in strategic locations (clinical skills labs, practical sessions, lecture halls)	☐ Yes ☐ No	
2	Running water is available during use	☐ Yes ☐ No	
3	Water faucet is easy to use and functions properly	☐ Yes ☐ No	
4	Soap dispenser is available	☐ Yes ☐ No	
5	Hand soap is available/provided	☐ Yes ☐ No	
6	Hand drying facilities are available (e.g., paper towels/dryer)	☐ Yes ☐ No	
7	Hand-wash station area is clean and odorless	☐ Yes ☐ No	
8	Drainage system functions properly (no clogging)	☐ Yes ☐ No	

B. Accessibility & Educational Support

No.	Observation Items	Checklist	Notes
1	Hand-wash station is easily accessible to students	☐ Yes ☐ No	
2	Instructional signs for hand-wash station usage are visible	☐ Yes ☐ No	
3	Handwashing educational posters/materials are displayed near the station	☐ Yes ☐ No	

Research Questionnaire

Instructions:
Please place a checkmark (✓) in the column that best corresponds to your opinion.
Likert Scale:

- **1** = Strongly Disagree (SD)
- **2** = Disagree (D)
- **3** = Agree (A)
- **4** = Strongly Agree (SA)

A. Handwashing Awareness (Based on the Health Belief Model)

Code	No.	Statement	SD (1)	D (2)	A (3)	SA (4)
**Perceived Benefits**	S1	Handwashing provides numerous benefits for my health.				
**Perceived Susceptibility**	S2	I feel vulnerable to contracting illnesses if I do not wash my hands after activities.				
**Perceived Severity**	S3	I consider handwashing to be a serious and critical action for maintaining my health.				
**Perceived Barriers**	S4	Difficulty accessing a hand-wash station hinders me from washing my hands.				
**Cues to Action**	S5	The presence of hand-wash stations around campus prompts me to be more aware of the importance of hand hygiene.				
**Self-Efficacy**	S6	I am confident in my ability to wash my hands correctly every day.				
**Perceived Benefits**	S7	I believe that handwashing can effectively prevent the transmission of diseases.				

B. Handwashing Compliance (Based on the Theory of Planned Behavior)

Code	No.	Statement	SD (1)	D (2)	A (3)	SA (4)
**Attitude Toward the Behavior**	P1	I have a well-established habit of washing my hands both before and after activities.				
**Attitude Toward the Behavior**	P2	I feel a sense of guilt if I fail to wash my hands after using the restroom.				

**Subjective Norms**	P3	My peers and lecturers wash their hands regularly, which motivates me to do the same.
**Perceived Behavioral Control**	P4	The available hand-wash facilities make it easy enough for me to practice hand hygiene.
**Actual Behavior (Outcome)**	P5	I perform a complete handwashing routine following the WHO 6-step technique.
**Actual Behavior (Outcome)**	P6	I wash my hands for a minimum of 20 seconds, as recommended by the WHO.

C. Hand-Wash Station Usage

Code	No.	Question	Yes	No
**G1**	1	Do you know the locations of the hand-wash stations across the campus environment?		
**G2**	2	In your opinion, are the campus hand-wash stations easily accessible?		
**G3**	3	Is the hand-wash station you typically use equipped with hand soap?		
**G4**	4	Is the campus hand-wash station you typically use equipped with hand drying facilities?		
**G5**	5	Have you ever used a campus hand-wash station to wash your hands?		
**G6**	6	Do you frequently use the hand-wash stations on campus?		
**G7**	7	Do you use the campus hand-wash stations before and after meals?		
**G8**	8	Do you use the campus hand-wash stations after using the restroom?		
**G9**	9	Would you continue to wash your hands at the station even if the water does not always flow smoothly?		
**G10**	10	Do you experience long queues or difficulties when attempting to use the campus hand-wash stations?		
**G11**	11	In your opinion, is the current number of permanent hand-wash stations sufficient to meet user needs?		
**G12**	12	In your opinion, are the campus hand-wash stations effective in supporting proper handwashing habits?		
**G13**	13	Are you familiar with the function and operational procedures of a surgical/sterile handwashing station (*scrub station*)?		
**G14**	14	Would the availability of a surgical/sterile handwashing station (*scrub station*) make it easier for you to wash your hands?		
